# Association of Obesity with Telomere Length in Human Sperm

**DOI:** 10.3390/jcm13072150

**Published:** 2024-04-08

**Authors:** Efthalia Moustakli, Athanasios Zikopoulos, Charikleia Skentou, Stefanos Dafopoulos, Sofoklis Stavros, Konstantinos Dafopoulos, Peter Drakakis, Ioannis Georgiou, Athanasios Zachariou

**Affiliations:** 1 Laboratory of Medical Genetics, Faculty of Medicine, School of Health Sciences, University of Ioannina, 45110 Ioannina, Greece; igeorgio@uoi.gr; 2 Obstetrics and Gynecology, Royal Devon and Exeter Hospital, Barrack Rd., Exeter EX 25 DW, UK; thanzik92@gmail.com; 3 Department of Obstetrics and Gynecology, Medical School of Ioannina, University General Hospital, 45110 Ioannina, Greece; haraskentou@gmail.com; 4 Department of Health Sciences, European University Cyprus, Nicosia 2404, Cyprus; stefanosntf2001@gmail.com; 5 Third Department of Obstetrics and Gynecology, Attikon Hospital, Medical School, National and Kapodistrian University of Athens, 12462 Athens, Greece; sfstavrou@med.uoa.gr (S.S.); pdrakakis@med.uoa.gr (P.D.); 6 IVF Unit, Department of Obstetrics and Gynecology, Faculty of Medicine, School of Health Sciences, University of Thessaly, 41500 Larissa, Greece; kdafop@yahoo.gr; 7 Department of Urology, School of Medicine, Ioannina University, 45110 Ioannina, Greece; zahariou@otenet.gr

**Keywords:** telomeres, telomere length, mitochondria, oxidation, body mass index, IVF/ICSI, male infertility, spermatozoa

## Abstract

**Background:** Telomere attrition and mitochondrial dysfunction are two fundamental aspects of aging. Calorie restriction (CR) is the best strategy to postpone aging since it can enhance telomere attrition, boost antioxidant capacity, and lower the generation of reactive oxygen species (ROS). Since ROS is produced by mitochondria and can readily travel to cell nuclei, it is thought to be a crucial molecule for information transfer between mitochondria and cell nuclei. Important variables that affect the quality and functionality of sperm and may affect male reproductive health and fertility include telomere length, mitochondrial content, and the ratio of mitochondrial DNA (mtDNA) to nuclear DNA (nDNA). Telomere damage results from mitochondrial failure, whereas nuclear DNA remains unaffected. This research aims to investigate potential associations between these three variables and how they might relate to body mass index. **Methods:** Data were collected from 82 men who underwent IVF/ICSI at the University Hospital of Ioannina’s IVF Unit in the Obstetrics and Gynecology Department. Evaluations included sperm morphology, sperm count, sperm motility, and participant history. To address this, male participants who were categorized into three body mass index (ΒΜΙ) groups—normal, overweight, and obese—had their sperm samples tested. **Results:** For both the normal and overweight groups, our results show a negative connection between relative telomere length and ΒΜI. As an illustration of a potential connection between mitochondrial health and telomere maintenance, a positive correlation was found for the obese group. Only the obese group’s results were statistically significant (*p* < 0.05). More evidence that longer telomeres are associated with lower mitochondrial content can be found in the negative connection between telomere length and mitochondrial content in both the normal and overweight groups. However, the obese group showed a positive association. The data did not reach statistical significance for any of the three groups. These associations may affect sperm quality since telomere length and mitochondrial concentration are indicators of cellular integrity and health. Moreover, the ratio of mtDNA to nDNA was positively correlated with the relative telomere lengths of the obese group, but negatively correlated with the normal and overweight groups. In every group that was studied, the results were not statistically significant. According to this, male fertility may be negatively impacted by an imbalance in the copy number of the mitochondrial genome compared to the nuclear DNA in sperm. **Conclusions:** Essentially, the goal of our work is to determine whether mitochondria and telomere length in human sperm interact. Understanding these connections may aid in the explanation of some male infertility causes and possibly contribute to the creation of new treatment modalities for problems pertaining to reproductive health. The functional implications of these connections and their applications in therapeutic settings require further investigation.

## 1. Introduction

The accumulation of abnormally high amounts of fat in the body is the hallmark of obesity. The obesogenic phenotype is driven by a confluence of hormonal, genetic, and metabolic factors that impact significant aspects of energy expenditure and nutritional intake [1]. Obesity accelerates aging-related pathologies including several cancer types, cardiovascular illnesses, type 2 diabetes (T2D), and non-alcoholic fatty liver disease (NAFLD), as well as organ degeneration [2,3,4]. Obesity causes early cellular senescence in a number of different tissues, which contributes to these difficulties, at least in part [5]. Obese adults exhibit telomere shortening, a defining feature of cellular senescence [6,7,8,9].

Cellular senescence is an irreversible cell cycle arrest caused by various types of stress. It can be triggered by DNA damage, telomere attrition, oxidative damage, mitotic stress, mitochondrial dysfunction, endoplasmic reticulum (ER) stress, and oncogene activation [10]. A process known as the mitochondrial dysfunction-associated senescent response (MiDAS) is a response that occurs when mitochondrial ROS and sirtuins are depleted. Hayflick and Moorhead first used the term “cellular senescence” to describe how human somatic cells gradually lose their ability to proliferate [11]. Along with the loss of the capacity to replicate, this condition also exhibits a variety of important modifications in cell structure, gene expression, metabοlism, epigenetics, and other areas [12]. The knowledge of how telomeres can signal senescence arrest has progressed significantly since then. These mechanisms are particularly crucial in the study of aging since it has been established that cellular senescence, which is fueled by telomere disruption, is a primary cause of aging and age-related disease [12].

Telomeres are DNA tandem repeats (TTAGGG) linked to a variety of proteins that together make up the “Shelterin” complex. At the end of linear chromosomes, there is a ribonucleoprotein called telomere repeats, which elongates the telomeric TTAGGG [13]. The core holoenzyme of this multi-subunit ribonucleoprotein enzyme only consists of catalytic telomerase reverse transcriptase (TERT) and an RNA template (TERC) [14]. Telomerase silencing occurs because TERC is widely expressed, while the TERT gene is strongly repressed in the majority of human somatic cells [15]. Hence, the factor that sets a rate limit for telomerase activity regulation is TERT. Telomeres have a 3′ overhang made up of single-stranded nucleotide repeats, as well as a lagging strand that is high in cytosine and a leading strand that is high in guanine [16]. Since continued cell division requires the maintenance of telomere length, telomerase is more active in cells that require this maintenance. Furthermore, telomerase’s reverse transcriptase structure is specifically made to withstand telomere shortening. In order to maintain ongoing cell division, which delays aging, the two major components of human telomerase—TERΤ and ΤΕRC—serve as templates for telomere extension. Telomerase activity increases with telomere length [17].

Extra-nuclear pools of the telomerase protein TERT are known to exist. Telomere independent functions were initially used to characterize TERT’s activities outside of the nucleus [18]. It is now well established that these activities are crucial for maintaining telomeres, maintaining genomic stability, and allowing interaction between the nucleus and mitochondria. TERT is released from the nucleus and imported into the mitochondria during conditions of stress, where it may have a protective role [19]. Furthermore, the TERC component is brought into the mitochondria, where it is converted to TERC-53 and subsequently released back to the cytosol, where it prevents GADPH from nuclear translocating [20,21]. Cytosolic TERC-53 is implicated in cellular senescence and organismal aging and transmits the signal of mitochondrial abnormalities to the nucleus. Genes implicated in the inflammatory response can also have their transcription increased by nuclear TERC binding directly to their promoter regions [22].

DNA breaks into single and double strands when the oxidative stress (OS) is high. Due to their higher guanine content and increased vulnerability to oxidative damage, telomeres also shorten more quickly as a result of it. The majority of somatic cells lack the telomerase enzyme, which results in a condition known as the “end-replication problem” during cell division. This is due to DNA polymerases’ innate inability to completely duplicate the lagging strand with a high concentration of telomere C [23]. By allowing DNA polymerases to start DNA replication, RNA primers contribute to the synthesis of the lagging strand. However, when the final primer at the 3′ end is deleted, telomere repeats are lost, and unavoidably, the recently formed strand will be a few nucleotides shorter [23].

It has been determined that the telomere length (TL) in human spermatozoa is roughly 6–20 kb, longer than that in somatic cells [24,25,26]. The arrangement of telomeres in sperm cells, specifically their location near the nuclear membrane or at the nuclear periphery, may have a purpose. This purpose is theorized to facilitate the oocyte’s access to these regions during fertilization, potentially influencing the growth of pronuclei. Another explanation for the differential distribution of telomeres concerning infertile males could be that poor telomere positioning makes the oocyte less accessible, which could be the cause of infertility. Telomere distribution in this case may suggest infertility, but it may also be the cause of infertility rather than just a symptom in an infertile man. Given that telomeres are histone-bound and positioned near nuclear boundaries, it is hypothesized that this may have a functional effect on the process of fertilization [27].

Telomeres, the protective caps at the ends of chromosomes, play a major role in cellular aging and senescence, in contrast to BMI, which is commonly used as a measure of general health and obesity. Mitochondrial DNA is essential for the cellular synthesis of energy and has implications for age-related disorders. The purpose of this research is to investigate any possible relationships between body mass index, mitochondrial function, and relative telomere length.

## 2. Materials and Methods

### 2.1. Participants’ Characteristics and Semen Sample Collection

The current study included eighty-two (82) men who underwent IVF/ICSI at the IVF Unit of the Obstetrics and Gynecology Department of the University Hοspital of Iοannina, Ioannina, Greece. Men were classified into three groups based on their BMI. Twenty-five [25] of the men recruited had normal ΒΜΙ (ranging from 18 to 24.9 kg/m^2^), twenty-nine [28] men were overweight (ranging from 25 to 29.9 kg/m^2^), and twenty-eight [29] men were obese (class I and II) with a ΒΜΙ higher than 30 kg/m^2^. Each participant signed an informed consent form. The characteristics of each sperm sample were identified using Kruger’s criteria.

According to Kruger’s criteria, tougher sperm morphology standards may be a reliable indicator of infertility during in vitro fertilization (IVF). The sperm head needs to meet Kruger’s specifications, which include having an oval form, regularity, and a smooth, continuous outline. Additionally, the acrosome must be intact. Furthermore, midpiece and tail abnormalities are not permitted. Another indication of morphological issues may be bent, numerous, or short tails as well as coiled, bowed, or sloppy midpieces. Finally, a high cytoplasmic droplet count may be a sign of sperm immaturity.

### 2.2. Sperm DNA Extraction and Quantification

In the fresh semen samples, sperm parameters such as sperm count, motility, morphology, and DFI using computer-aided semen analysis (CASA), were measured within an hour of ejaculation. The Sperm Class Analyzer (SCA) software SCA 6 Evolution from MICROPTIC Automatic Diagnostic Systems SL (Barcelona, Spain), an Axio Zeiss^®^ Lab.A1 microscope, and a Basler Ace digital recording camera (Jena, Germany), were used to measure concentration and motility. The samples were then frozen at a temperature of –20 °C for storage. The Qiagen (Venlo, The Netherlands) methodology was used in the current investigation along with a matching kit, the QIAmp DNA blood Mini kit by Qiagen. During the first stage, 100 μL of semen sample was combined with an X2 buffer, and heated for at least an hour at 56 °C in the heat block. Each sample was vortexed at regular intervals. Following this, a volume of 200 μL from each preheated semen sample was processed for sperm DNA extraction using the QIAmp DNA blood Mini kit according to the manufacturer’s instructions.

Using a NanoDrop Spectrophotometer from Thermo Fisher Scientific, Waltham, MA, USA, the isolated sperm DNA was eluted in 80 μL of elution buffer and measured to determine purity and concentrations. By evaluating the A260/A80 ratio using spectroscopy for DNA analysis, the quality of the isolated DNA was evaluated. A human cell population’s average telomere length can be measured directly using the Absolute Human Telomere Length Quantification qPCR Assay Kit (AHTLQ) from ScienCell Research Laboratories, San Diego, California. Recοgnizing and amplifying telοmere sequences is the function of the telomere primer pair. Data normalization was accomplished using the single-copy reference (SCR) primer pair, which identifies and amplifies the human chromosome 17 region of 100 base pairs.

Two genes were chosen for DNA quantification, both nuclear and mitochondrial DNA. The human retinoid isomerohydrolase gene (RPE65) served as the nuclear DNA reference gene for the quantification of nDNA using the RPE primers 5′-ATAGGAAGCCAGAGAAGAGAGACT-3′ and 5′-TCTATCTCTGCGGACTTTGAGCAT-3′ (200 bp). Meanwhile, the combination of primers 5′-TAGAGGAGCCTGTTCTGTAATCG-3′ and 5′-TAAGGGCTATCGTAGTTTTCTGG-3′ (205 bp) was utilized for the mitochondrial DNA quantification, which corresponds to a section of the mitochondrially produced 12S RNA (Table 1).

### 2.3. Real-Time Quantitive PCR (qPCR)

A quantitative polymerase chain reaction was employed to measure the absolute telomere length. Every genomic DNA sample was used in two separate qPCR procedures, one using telomere primer stock solution and the other using SCR primer stock solution. We employed the following components in each reaction: 0.5–0.5 ng/μL of genomic DNA template, 2 μL of primer stock solution (Telomere or SCR), 10 μL of 2X GoldNStart TaqGreen qPCR master mix, and 7 μL of nuclease-free water, for a total volume of 20 μL. The reaction was conducted on a Corbett Rotor-Gene 3000 Real-Time Rotary Analyzer (Corbett Research, Sydney, Australia) using the following cycling conditions: initial denaturation at 95 °C for 10 min, followed by 32 cycles of denaturing at 95 °C for 20 s, annealing at 52 °C for 20 s, and extension at 72 °C for 45 s. Following data collection, a hold at 25 °C for 1 min was performed.

### 2.4. Quantification Method: Comparative ΔΔCq Method Based on the AHTLQ Assay Kit

1. The difference in the number of quantification cycles of telomeres (TEL) between the target and reference genomic DNA samples is known as Cq (TEL).

ΔCq (TEL) = Cq (TEL, target sample)—Cq (TEL, reference sample)

2. For single-copy references, Cq (SCR) is the number of quantification cycles that differ between the reference and target genomic DNA samples.

ΔΔCq = ΔCq (TEL) – ΔCq (SCR)

3. The target sample’s relative telomere length to the reference sample (fold) = 2^−ΔΔCq^.

The mitochondrial DNA content and the mtDNA to nDNA ratio were evaluated using quantitative polymerase chain reaction (qPCR). The following ingredients were added to each reaction: 0.02–0.03 μg of extracted DNA, 0.25 μL of each 10 μM forward and reverse primer, 5 μL of SYBR Green Master Mix (PowerUp SYBR Green Master Mix, Applied Biosystems, Thermo Fisher Scientific, USA), and 2.5 μL of distilled water, for a total volume of 10 μL. The following cycling conditions were used for the reaction on a Corbett Rotor-Gene 3000 Real-Time Rotary Analyzer (Corbett Research, Sydney, Australia): 50 °C for 2 min, 95 °C for 2 min, and then 40 cycles of denaturing at 95 °C for 15 s and 60 °C for 1 min. A duplicate of each sample was analyzed. The proportion of mitochondrial DNA in each sperm was measured. After calculating the ratio of mtDNA to nDNA using the ΔΔCt technique—which is described below—the relative mitochondrial DNA content per sperm sample was determined.

Selecting a control group, we used the cycle threshold Ct values for the relative quantification (also referred to as the “ΔΔCt method” as follows [28,29]:

(a) By subtracting each sample’s mean nuclear Ct value from its mean mitochondrial Ct value, as shown by the formula ΔCt = mtDNA – nDNA;
(b) In this case, the control group comprised men with normal body mass index (18.5 to 24.9 kg/m^2^) and normal semen characteristics, that is, the average ΔCt value;
(c) A sample’s ΔΔCt is determined by deducting the control group’s ΔCt from the sample’s mean ΔCt. In other words, ΔΔCt = a sample’s ΔCt minus the control group’s ΔCt [28];
(d) The fold difference is calculated using the formula 2^−ΔΔCt^.

### 2.5. Statistical Analysis

The association between the two variables was evaluated statistically using Spearman’s correlation coefficient. The statistical software SPSS 28.0 was used to analyze the data between the groups under study. The direction and correlation coefficient (r_s_) between the variables shown in the graph are reported numerically in the analysis. Microsoft Excel spreadsheets were used to organize the extraction data, mtDNA copy numbers, and Ct rates to create graphs. Statistical significance was defined as *p* < 0.05.

### 2.6. Inclusion and Exclusion Criteria

The inclusion and exclusion criteria that were taken into consideration for the conduct of the particular study are referenced in the following table (Table 2).

## 3. Results

The individuals were divided into three groups according to their BMI. The mean values for the males’ semen characteristics are displayed in the table below (Table 3).

Furthermore, the median values of the relative length of the telomeres for the three groups under study are reported in the graph below.

The overweight group’s TL differs by almost half from that of the groups with normal and obese BMIs. However, this difference is not significant in terms of statistics (*p* > 0.05).

**Figure 1:**
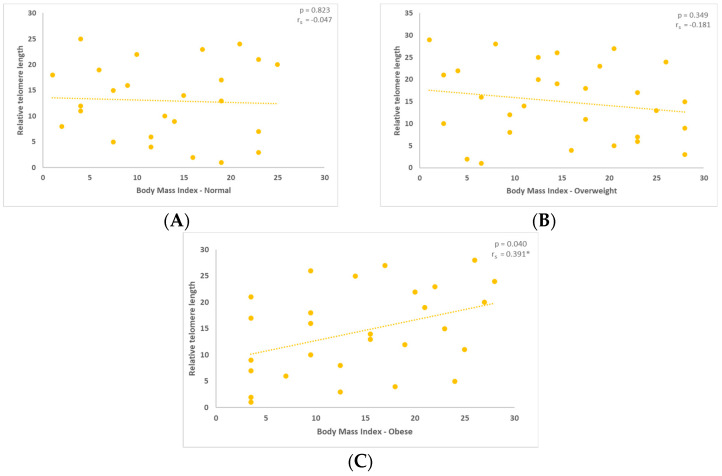
(**A**,**B**) Evaluation of the relative telomere length (TL) in the normal and overweight groups. The outcomes were not statistically significant with such *p* values, 0.823 and 0.349, respectively. (**C**) With a statistical significance level of 0.040, the measurement of the relative TL in the obese group revealed a positive association (correlation coefficient r_s_ = 0.391 *; is significant at the 0.05 level, two-tailed).

Following that, in each group separately, we examined the interaction regarding relative telomere length (TL). For the three groups with normal, overweight, and obese BMI, the relative length of telomeres was examined, as shown in the figures below. A substantial negative association was found between telomere length (TL) and the group of people with normal BMI (Figure 1A).

**Figure A1:**
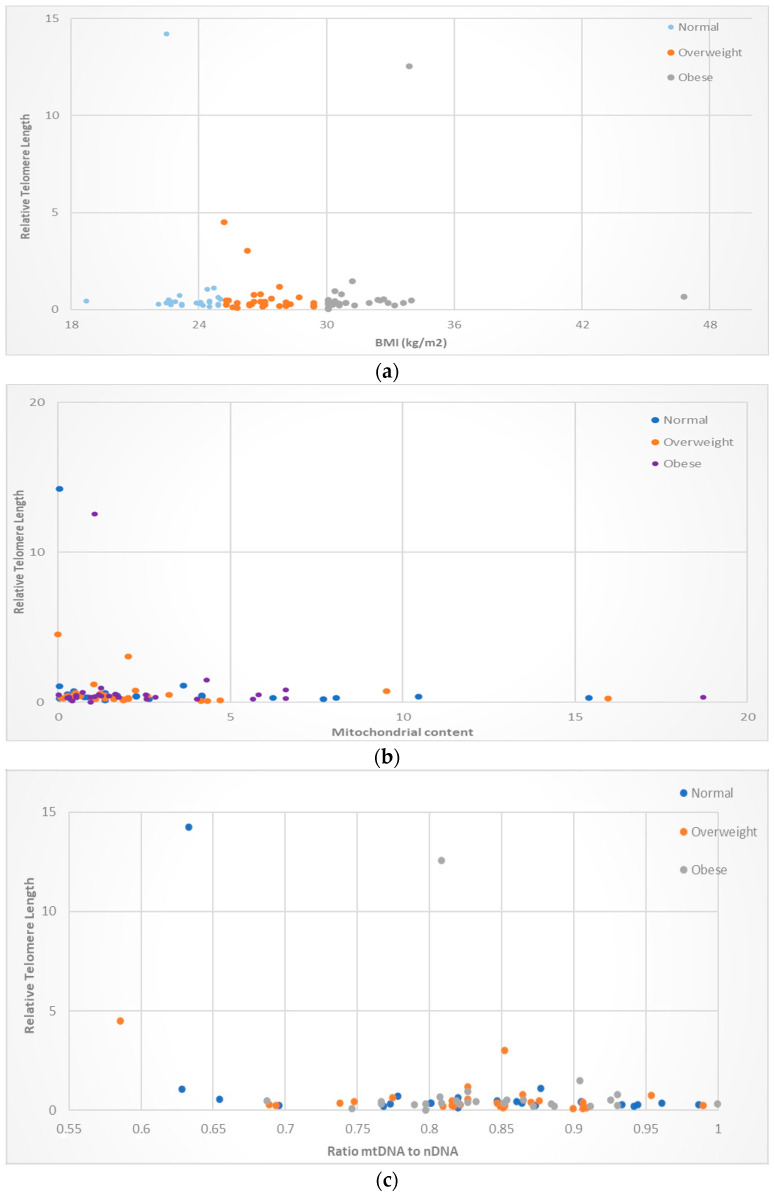
A comprehensive representation of the variables examined in this research. (**a**) Body mass index-based variation in relative telomere length. (**b**) Distribution of the mitochondrial content based on the relative telomere length. (**c**) Correlation between nuclear to mitochondrial DNA ratio and relative telomere length.

A negative association between BMI and relative TL was also observed in the overweight group, as indicated in the following graph (Figure 1B). Finally, relative TL demonstrated a positive correlation with BMI in the obese group (Figure 1C, Figure A1a). The figures below depict the results of the Spearman’s correlation coefficient conducted to examine the relationship between relative TL and BMI.

### 3.1. Correlation between Mitochondrial DNA Content and the Relative Telomere Length

**Figure 2:**
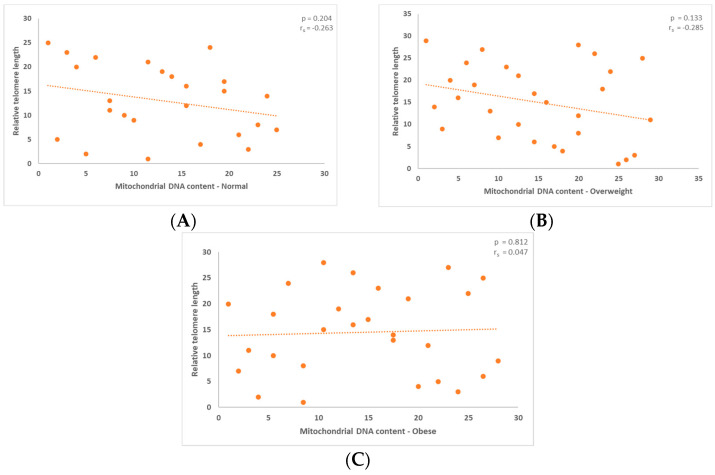
(**A**,**B**) For men in the normal and overweight groups, relative telomere length and mitochondrial DNA content did not show any statistically significant outcomes, *p* = 0.204 and *p* = 0.133, respectively. (**C**) In the obese group, relative TL and mitochondrial DNA load did not show any statistical significance, *p* = 0.812.

Additionally, the normal and overweight groups’ correlations between mitochondrial content and TL revealed a negative association. The results, which are shown in the figures below, are not statistically significant, as indicated by the *p*-values. The *p*-value for the normal group was 0.204 (Figure 2A), while it was 0.183 for the overweight group (Figure 2B).

The comparison of mitochondrial content and relative telomere length for males who range into the obese category showed a positive connection. The graph below reflects this further (Figure 2C, Figure A1b). The outcome in this instance was statistically significant, *p* = 0.812.

### 3.2. Correlation between mtDNA-to-nDNA Ratio and the Relative Telomere Length

**Figure 3:**
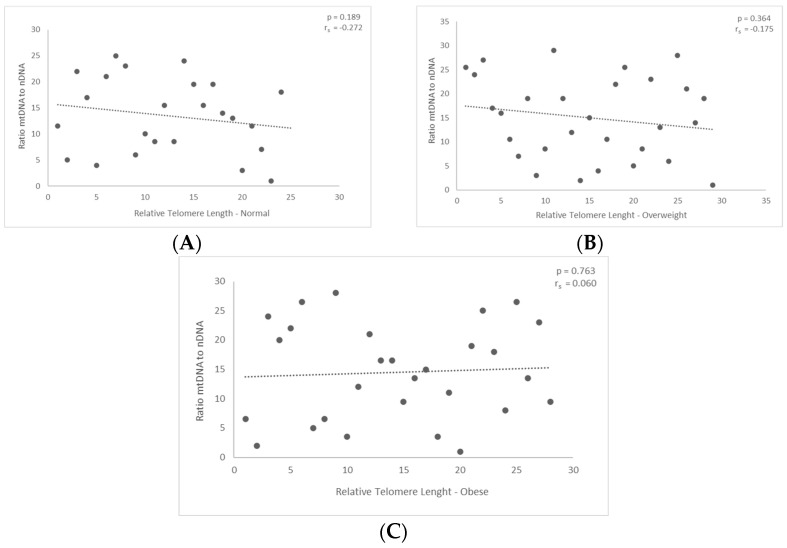
(**A**,**B**) Males with normal and overweight BMI showed no statistically significant differences in the ratio of mitochondrial to nuclear DNA with relative telomere length. (**C**) The ratio of mitochondrial to nuclear DNA and the relative telomere length of obese males indicates that the results are not statistically significant.

Next, we assessed the correlation between relative telomere length and the ratio of nuclear to mitochondrial DNA. The diagram demonstrates the first finding, which is that there was a negative correlation between those with normal and overweight BMIs. The findings (*p* = 0.189 and *p* = 0.36, respectively) did not demonstrate any statistical significance (Figure 3A and Figure 3B, respectively).

Nevertheless, the outcomes did not reach statistical significance for the group classified as obese. In particular, the ratio displayed a modest positive correlation with the relative length of telomeres in the obese group; however, the *p*-value is only *p* = 0.763 (Figure 3C, Figure A1c). The results obtained were not statistically significant.

## 4. Discussion

Obesity has long been thought to have a deleterious impact on male fertility [30]. The precise processes and consequences of these instances on male fertility remain unclear despite significant advancements in the research. Consequently, this study offered a novel viewpoint on the connection between male fertility and mitochondria, telomere length, and body mass index. Initially, the relationship between relative telomere length and body mass index was investigated. It was found that the only group with a positive correlation and statistically significant results was the obese group, suggesting that weight affects telomere length and consequently sperm quality. Regarding the normal and overweight categories, no association was noticed.

Additionally, we noted that the obese group’s average relative telomere length was lower than that of the normal group. The overweight population is covered by this as well. However, the average relative telomere length was higher in the obese group than in the overweight group. This result is consistent with a different study that used an adequate number of participants [31]. While we recruited patients with obesity (BMI ≥ 30 kg/m^2^) from the general population and not just the infertile category, the previously mentioned study recruited patients from infertile couples undergoing their first fresh IVF cycle with male BMI of more than 28 (which includes both overweight and obese patients) [31]. Consideration should be given to a complex mechanism comprising confounding variables, as telomere shortening in obese individuals may not be a direct result of obesity. Weight-related OS and increased ROS levels, which cause telomere shortening, could be one of the main reasons for this [32].

Telomeres, telomerase subunits, and mitochondria have been intimately linked in recent years. Telomerase’s primary function is to prevent telomere shortening and preserve telomere length. Furthermore, telomeres are involved in maintaining cell viability. It has been discovered that telomerase is the enzyme that catalyzes the removal of the oxidative stress-stimulated subunit TERT from the nuclei and deposits it in the mitochondria. By lowering mitochondrial ROS generation, this co-localization of TERT and mitochondria maintains mitochondrial function and mitigates nuclear DNA damage and apoptosis [33,34]. Interestingly, there is either no or very little DNA damage in cells that entirely eliminate telomerase. On the other hand, telomerase levels in cells rise in response to damage to cellular DNA that has not been entirely eliminated. Telomerase, according to the authors, may negatively impact cell nuclei’s repair enzymes, potentially leading to increased DNA damage [35]. This also clarifies why, to some extent, telomerase is unable to resume its function following DNA damage.

Telomere damage results in the reprogramming of mitochondrial biosynthesis and mitochondrial dysfunction, which have significant consequences for aging and diseases. Nonetheless, mitochondrial dysfunction causes telomere attrition [18]. In our study, the findings obtained when comparing mitochondrial content and relative telomere length based on BMI were not statistically significant. However, it appears that there is a positive correlation for the obese group and a negative correlation for the normal and overweight groups. Lastly, we investigated the relationship between the ratio of mitochondrial DNA to nuclear DNA and the relative telomere length for each group of participants. The correlation was positive for the obese group and negative for the normal and overweight groups. However, for all the groups under investigation (normal, overweight, and obese), neither case showed statistically significant outcomes.

Telomere shortening and mitochondrial dysfunction are features of cellular senescence. They are tightly associated, with the former influencing mitochondrial function through p53-PGC-1α signaling pathways. While aging is unavoidable, growing older may shorten life expectancy due to elevated risk factors for chronic diseases. Different studies suggest that telomere attrition is modifiable, as substantial variability exists in the rate of telomere shortening that is independent of chronological age [36]. Telomere attrition has also been linked with other potentially modifiable lifestyle factors, such as poor nutrition and physical inactivity, indicating the plasticity of TL [37,38]. Findings from different studies show that a healthy diet, low stress, exercise, and a good sleep pattern are related to longer telomeres [39,40]. In the absence of direct evidence, the positive correlation between sperm telomere length (STL) and sperm DNA fragmentation in young individuals may be due to the activation of telomerase by a low-level adverse factor, such as mild OS, which causes genomic damage in addition to STL extension [41,42,43].

The normal process of fertilization may be affected by alterations in the mitochondrial DNA (mtDNA) [44,45,46,47]. Nonetheless, not much research has been conducted on potential correlations between telomeres and mitochondrial characteristics in human sperm. It is widely acknowledged that mtDNA is especially prone to cumulative mutation and deletion, either as a result of its proximity to the respiratory chains, which produce ROS, or because it lacks protective histones [48]. Oxidative insults are known to affect both nuclear and mitochondrial DNA [49]. A body of evidence has been gathered demonstrating that telomerase can also function in telomere-independent ways and that, under OS, the TERT transfers between the nucleus and mitochondria, and the TERC is processed, imported into mitochondria, and exported back to the cytosol [18].

Telomere length and structure are essential for maintaining the integrity of the nuclear genome, and spermatogenesis and conception are specifically linked to the maintenance of telomeres in germ cells [50]. An essential component of the dynamic maintenance of telomere length homeostasis is the existence of telomerase activity in spermatogenic cells. Previous research, however, has demonstrated that both endogenous and exogenous variables, including OS, inflammation, exposure to the environment, and lifestyle choices, may disrupt telomere homeostasis [51,52,53,54]. According to several studies, telomerase may even be triggered in some circumstances, leading to telomere elongation [51,52,53]. Low levels of arsenic exposure and oxidative stress have both been shown to boost telomerase activity and lengthen telomeres [54,55,56]. Given that early research is starting to reveal associations between sperm TL and reproductive outcomes, TL is a desirable topic for further research. Future research on these topics should be conducted in depth.

## 5. Conclusions

This investigation into the relationship between relative TL and mitochondrial DNA content has shed light on the complex interactions between these two vital aspects of cellular health and aging [49]. The findings highlight the significance of preserving mitochondrial health and telomere integrity as critical elements in promoting lifespan and general well-being, even though further study is required to clarify the specific nature of this link. This discovery could influence future medical therapies and methods meant to maintain cellular vitality and lengthen healthy lifespans as our understanding of these interrelated processes continues to advance.

## 6. Limitations of This Study

When evaluating this study’s findings, one should take into account a number of its shortcomings. Firstly, a smaller sample size may have limited how broadly the results may be applied to the public. Furthermore, answer bias and recall errors may have been introduced by the data collection strategy, which mostly relied on self-report surveys. This study was also limited by the geographic area in which it was carried out; thus, contextual and cultural factors may have a different effect on the findings elsewhere. Relative measurements are often obtained by PCR-based methods, which are the most widely used approaches for measuring mtDNA-CN and telomere length. Furthermore, no research has been conducted that focuses on population-based studies and systematically examines the relationship between telomere length, mtDNA-CN, and sperm prevalence, incidence, and death. Finally, logistical and ethical limitations may have affected this study’s methods and results, as they do with any research involving human beings. These restrictions highlight the need for additional investigation to strengthen and improve the understanding that this study has provided.

## Figures and Tables

**Table 1:** Primer sequences, amplicon base pair, and Tm.

Primer Pair	Sequence (Forward/Reverse)	Amplicon Base Pair (bp)	Tm
Nuclear region (RPE gene)	Forward 5′-ATAGGAAGCCAGAGAAGAGAGACT-3′ Reverse 5′-TCTATCTCTGCGGACTTTGAGCAT-3′	200	60 °C
Mitochondrial region	Forward 5′-TAGAGGAGCCTGTTCTGTAATCG-3′ Reverse 5′-TAAGGGCTATCGTAGTTTTCTGG-3′	205	59 °C

**Table 2:** Inclusion and exclusion criteria for this study.

Inclusion Criteria	Exclusion Criteria
IVF/ICSI participants	Males without informed consent
Lifestyle (smoking, alcohol, drugs, etc.)	Urological conditions (e.g., varicocele)
	Abnormal Karyotype
	AZF deletions

**Table 3:** Male participants’ age, morphology, progressive motility, and sperm count, with a 95% confidence interval (CI), were among the parameters of their semen that were examined in this study. *p* values were determined by ANOVA. *p* < 0.05 was used to indicate statistical significance.

Semen Characteristics	Normal	Overweight	Obese	*p*-Values
No. of participants	25	29	28	
Progressive motility (%), CI 95%	47.71 ± 6.14	37.53 ± 5.61	4.2 ± 3.56	0.0062
Sperm morphology (%), CI 95%	13.48 ± 4.11	16 ± 9.04	10.16 ± 3.02	0.4073
Sperm count, million/mL, CI 95%	30.54 ± 14.84	16.98 ± 8.74	34.56 ± 12.67	0.0692
Male age, CI 95%	38.3 ± 1.75	43.25 ± 2.75	41 ± 2.55	0.022

## Data Availability

Data are unavailable due to privacy or ethical restrictions.

## Appendix A

We offer a thorough summary of all the variables examined in this research in this appendix. To improve transparency and reproducibility for readers and other researchers, we hope that this information will be presented in an orderly and systematic way, enabling a greater understanding of the variables and their impact on our study.

## References

[1] Cheng L., Wang J., Dai H., Duan Y., An Y., Shi L., Lv Y., Li H., Wang C., Ma Q. (2021). Brown and beige adipose tissue: A novel therapeutic strategy for obesity and type 2 diabetes mellitus. Adipocyte.

[2] Kawai T., Autieri M.V., Scalia R. (2021). Adipose tissue inflammation and metabolic dysfunction in obesity. Am. J. Physiol.-Cell Physiol..

[3] Roberto C.A., Swinburn B., Hawkes C., Huang T.T.-K., Costa S.A., Ashe M., Zwicker L., Cawley J.H., Brownell K.D. (2015). Patchy progress on obesity prevention: Emerging examples, entrenched barriers, and new thinking. Lancet.

[4] Santos A.L., Sinha S. (2021). Obesity and aging: Molecular mechanisms and therapeutic approaches. Ageing Res. Rev..

[5] Burton D.G.A., Faragher R.G.A. (2018). Obesity and type-2 diabetes as inducers of premature cellular senescence and ageing. Biogerontology.

[6] Welendorf C., Nicoletti C.F., Pinhel M.A.D.S., Noronha N.Y., De Paula B.M.F., Nonino C.B. (2019). Obesity, weight loss, and influence on telomere length: New insights for personalized nutrition. Nutrition.

[7] García-Calzón S., Moleres A., Marcos A., Campoy C., Moreno L.A., Azcona-Sanjulián M.C., Martínez-González M.A., Martínez J.A., Zalba G., Marti A. (2014). Telomere length as a biomarker for adiposity changes after a multidisciplinary intervention in overweight/obese adolescents: The EVASYON study. PLoS ONE.

[8] Lee M., Martin H., Firpo M.A., Demerath E.W. (2011). Inverse association between adiposity and telomere length: The Fels Longitudinal Study. Am. J. Hum. Biol. Off. J. Hum. Biol. Counc..

[9] Rode L., Nordestgaard B.G., Weischer M., Bojesen S.E. (2014). Increased Body Mass Index, Elevated C-Reactive Protein, and Short Telomere Length. J. Clin. Endocrinol. Metab..

[10] Narasimhan A., Flores R.R., Camell C.D., Bernlohr D.A., Robbins P.D., Niedernhofer L.J. (2022). Cellular Senescence in Obesity and Associated Complications: A New Therapeutic Target. Curr. Diab. Rep..

[11] Hayflick L., Moorhead P.S. (1961). The serial cultivation of human diploid cell strains. Exp. Cell Res..

[12] van Deursen J.M. (2014). The role of senescent cells in ageing. Nature.

[13] Bhari V.K., Kumar D., Kumar S., Mishra R. (2021). Shelterin complex gene: Prognosis and therapeutic vulnerability in cancer. Biochem. Biophys. Rep..

[14] Yuan X., Xu D. (2019). Telomerase Reverse Transcriptase (TERT) in Action: Cross-Talking with Epigenetics. Int. J. Mol. Sci..

[15] Yuan X., Larsson C., Xu D. (2019). Mechanisms underlying the activation of TERT transcription and telomerase activity in human cancer: Old actors and new players. Oncogene.

[16] Griffith J.D., Comeau L., Rosenfield S., Stansel R.M., Bianchi A., Moss H., De Lange T. (1999). Mammalian Telomeres End in a Large Duplex Loop. Cell.

[17] Vaiserman A., Krasnienkov D. (2021). Telomere Length as a Marker of Biological Age: State-of-the-Art, Open Issues, and Future Perspectives. Front. Genet..

[18] Zheng Q., Huang J., Wang G. (2019). Mitochondria, Telomeres and Telomerase Subunits. Front. Cell Dev. Biol..

[19] Green P.D., Sharma N.K., Santos J.H. (2019). Telomerase Impinges on the Cellular Response to Oxidative Stress Through Mitochondrial ROS-Mediated Regulation of Autophagy. Int. J. Mol. Sci..

[20] Huang J., Liu P., Wang G. (2018). Regulation of mitochondrion-associated cytosolic ribosomes by mammalian mitochondrial ribonuclease T2 (RNASET2). J. Biol. Chem..

[21] Cheng Y., Liu P., Zheng Q., Gao G., Yuan J., Wang P., Huang J., Xie L., Lu X., Tong T. (2018). Mitochondrial Trafficking and Processing of Telomerase RNA TERC. Cell Rep..

[22] Liu H., Yang Y., Ge Y., Liu J., Zhao Y. (2019). TERC promotes cellular inflammatory response independent of telomerase. Nucleic Acids Res..

[23] Chan S.R.W.L., Blackburn E.H. (2004). Telomeres and telomerase. Philos. Trans. R. Soc. Lond. B. Biol. Sci..

[24] Kaltsas A., Moustakli E., Zikopoulos A., Georgiou I., Dimitriadis F., Symeonidis E.N., Markou E., Michaelidis T.M., Tien D.M.B., Giannakis I. (2023). Impact of Advanced Paternal Age on Fertility and Risks of Genetic Disorders in Offspring. Genes.

[25] Kimura M., Cherkas L.F., Kato B.S., Demissie S., Hjelmborg J.B., Brimacombe M., Cupples A., Hunkin J.L., Gardner J.P., Lu X. (2008). Offspring’s leukocyte telomere length, paternal age, and telomere elongation in sperm. PLoS Genet..

[26] Baird D.M., Britt-Compton B., Rowson J., Amso N.N., Gregory L., Kipling D. (2006). Telomere instability in the male germline. Hum. Mol. Genet..

[27] Turner K.J., Watson E.M., Skinner B.M., Griffin D.K. (2021). Telomere Distribution in Human Sperm Heads and Its Relation to Sperm Nuclear Morphology: A New Marker for Male Factor Infertility?. Int. J. Mol. Sci..

[28] Quiros P.M., Goyal A., Jha P., Auwerx J. (2017). Analysis of mtDNA/nDNA Ratio in Mice. Curr. Protoc. Mouse Biol..

[29] Livak K.J., Schmittgen T.D. (2001). Analysis of relative gene expression data using real-time quantitative PCR and the 2(-Delta Delta C(T)) Method. Methods San Diego Calif.

[30] Zia S. (2023). Obesity: Impact and Outcome on Infertility—A Literature Review. Open J. Obstet. Gynecol..

[31] Yang Q., Zhao F., Hu L., Bai R., Zhang N., Yao G., Sun Y. (2016). Effect of paternal overweight or obesity on IVF treatment outcomes and the possible mechanisms involved. Sci. Rep..

[32] Raee P., Shams Mofarahe Z., Nazarian H., Abdollahifar M.-A., Ghaffari Novin M., Aghamiri S., Ghaffari Novin M. (2023). Male obesity is associated with sperm telomere shortening and aberrant mRNA expression of autophagy-related genes. Basic Clin. Androl..

[33] Qi H., Chen Y., Fu X., Lin C.-P., Zheng X.F.S., Liu L.F. (2008). TOR Regulates Cell Death Induced by Telomere Dysfunction in Budding Yeast. PLoS ONE.

[34] Ahmed S., Passos J.F., Birket M.J., Beckmann T., Brings S., Peters H., Birch-Machin M.A., von Zglinicki T., Saretzki G. (2008). Telomerase does not counteract telomere shortening but protects mitochondrial function under oxidative stress. J. Cell Sci..

[35] Singhapol C., Pal D., Czapiewski R., Porika M., Nelson G., Saretzki G.C. (2013). Mitochondrial Telomerase Protects Cancer Cells from Nuclear DNA Damage and Apoptosis. PLoS ONE.

[36] Du M., Prescott J., Kraft P., Han J., Giovannucci E., Hankinson S.E., De Vivo I. (2012). Physical Activity, Sedentary Behavior, and Leukocyte Telomere Length in Women. Am. J. Epidemiol..

[37] Cassidy A., De Vivo I., Liu Y., Han J., Prescott J., Hunter D.J., Rimm E.B. (2010). Associations between diet, lifestyle factors, and telomere length in women. Am. J. Clin. Nutr..

[38] Puterman E., Lin J., Blackburn E., O’Donovan A., Adler N., Epel E. (2010). The Power of Exercise: Buffering the Effect of Chronic Stress on Telomere Length. PLoS ONE.

[39] Farzaneh-Far R. (2010). Association of Marine Omega-3 Fatty Acid Levels with Telomeric Aging in Patients with Coronary Heart Disease. JAMA.

[40] Ornish D., Lin J., Daubenmier J., Weidner G., Epel E., Kemp C., Magbanua M.J.M., Marlin R., Yglecias L., Carroll P.R. (2008). Increased telomerase activity and comprehensive lifestyle changes: A pilot study. Lancet Oncol..

[41] Wyrobek A.J., Eskenazi B., Young S., Arnheim N., Tiemann-Boege I., Jabs E.W., Glaser R.L., Pearson F.S., Evenson D. (2006). Advancing age has differential effects on DNA damage, chromatin integrity, gene mutations, and aneuploidies in sperm. Proc. Natl. Acad. Sci. USA.

[42] Vagnini L., Baruffi R.L.R., Mauri A.L., Petersen C.G., Massaro F.C., Pontes A., Oliveira J.B.A., Franco J.G. (2007). The effects of male age on sperm DNA damage in an infertile population. Reprod. Biomed. Online.

[43] Gunes S., Hekim G.N.T., Arslan M.A., Asci R. (2016). Effects of aging on the male reproductive system. J. Assist. Reprod. Genet..

[44] Kim J.-H., Kim H.K., Ko J.-H., Bang H., Lee D.-C. (2013). The relationship between leukocyte mitochondrial DNA copy number and telomere length in community-dwelling elderly women. PLoS ONE.

[45] Qiu C., Enquobahrie D.A., Gelaye B., Hevner K., Williams M.A. (2015). The association between leukocyte telomere length and mitochondrial DNA copy number in pregnant women: A pilot study. Clin. Lab..

[46] Alegría-Torres J.A., Velázquez-Villafaña M., López-Gutiérrez J.M., Chagoyán-Martínez M.M., Rocha-Amador D.O., Costilla-Salazar R., García-Torres L. (2016). Association of Leukocyte Telomere Length and Mitochondrial DNA Copy Number in Children from Salamanca, Mexico. Genet. Test. Mol. Biomark..

[47] Melicher D., Illés A., Littvay L., Tárnoki Á.D., Tárnoki D.L., Bikov A., Kunos L., Csabán D., Buzás E.I., Molnár M.J. (2021). Positive association and future perspectives of mitochondrial DNA copy number and telomere length—A pilot twin study. Arch. Med. Sci. AMS.

[48] Giorgi C., Marchi S., Simoes I.C.M., Ren Z., Morciano G., Perrone M., Patalas-Krawczyk P., Borchard S., Jędrak P., Pierzynowska K. (2018). Mitochondria and Reactive Oxygen Species in Aging and Age-Related Diseases. Int. Rev. Cell Mol. Biol..

[49] Moustakli E., Zikopoulos A., Sakaloglou P., Bouba I., Sofikitis N., Georgiou I. (2023). Functional association between telomeres, oxidation and mitochondria. Front. Reprod. Health.

[50] Fice H., Robaire B. (2019). Telomere Dynamics throughout Spermatogenesis. Genes.

[51] Yim H.W., Slebos R.J.C., Randell S.H., Umbach D.M., Parsons A.M., Rivera M.P., Detterbeck F.C., Taylor J.A. (2007). Smoking is associated with increased telomerase activity in short-term cultures of human bronchial epithelial cells. Cancer Lett..

[52] Ferrario D., Collotta A., Carfi M., Bowe G., Vahter M., Hartung T., Gribaldo L. (2009). Arsenic induces telomerase expression and maintains telomere length in human cord blood cells. Toxicology.

[53] Mo J., Xia Y., Ning Z., Wade T.J., Mumford J.L. (2009). Elevated human telomerase reverse transcriptase gene expression in blood cells associated with chronic arsenic exposure in Inner Mongolia, China. Environ. Health Perspect..

[54] López-Diazguerrero N.E., Pérez-Figueroa G.E., Martínez-Garduño C.M., Alarcón-Aguilar A., Luna-López A., Gutiérrez-Ruiz M.C., Königsberg M. (2012). Telomerase activity in response to mild oxidative stress. Cell Biol. Int..

[55] Wang Z., Rhee D.B., Lu J., Bohr C.T., Zhou F., Vallabhaneni H., de Souza-Pinto N.C., Liu Y. (2010). Characterization of oxidative guanine damage and repair in mammalian telomeres. PLoS Genet..

[56] Mishra S., Kumar R., Malhotra N., Singh N., Dada R. (2016). Mild oxidative stress is beneficial for sperm telomere length maintenance. World J. Methodol..

